# Supplementary material for: Modeling attention and binding in the brain through bidirectional recurrent gating
Source: Nat Commun. 2026 May 5;17:4072. doi: 10.1038/s41467-026-72146-9 (PMC13144683; doi:10.1038/s41467-026-72146-9)
Supplement: Supplementary file 1 — Supplementary Information [file 41467_2026_72146_MOESM1_ESM.pdf]

Supplementary material for
Modeling Attention and Binding in the Brain through Bidirectional Recurrent Gating

Saeed Salehi, Jordan Lei, Ari Benjamin, Klaus-Robert Müller, Konrad Kording

Single-task versus multitask training

To deepen our understanding of the multitask framework, we conducted three additional sets of experiments designed to explicitly evaluate the effects of multitask learning on performance, interference/facilitation dynamics, shared representations, and scalability. These include: (1) A comparison between multitask and single-task models (Fig. 1); (2) Transfer learning from single-task pretraining to a multitask setting (Figs. 3, 4); and (3) Representational Similarity Analysis to examine the emergence of shared representations (Figs. 5, 6). Together, these experiments offer a more comprehensive view of the strengths and limitations of our multitask learning paradigm.

Multitask versus single-task learning

We performed single-task training for all tasks in both the MNIST and COCO families to evaluate whether the network could solve each task independently, and to assess the impact of multitask learning on cross-task generalization. We used the same architecture across all MNIST-based tasks, and similarly across all COCO-based tasks. Training epochs were adjusted to ensure that each single-task model received a comparable number of updates to the multitask model. Hyperparameters were kept consistent within each task family, with the exception of the symbolic orienting task, where the learning rate was reduced to 0.0001 to facilitate convergence (Fig. 1).

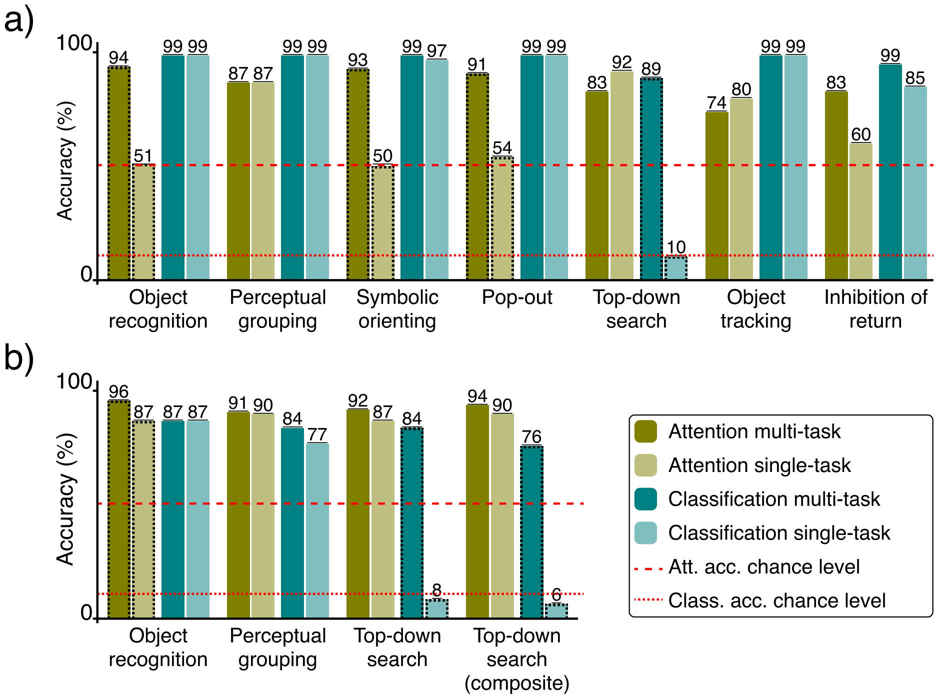

Supplementary Figure. 1. Multitask versus single-task learning. a) Results for the MNIST family. The model performs equally well on single-tasks that receive both classification and attention error signals during training (e.g. the perceptual grouping task) compared to the multitask training. However, for the partially supervised tasks such as object recognition and top-down search, the single-task model achieves high accuracy on the supervised component but does not match the performance of the multitask model. **b)** Results for the COCO task family, showing similar trends. Dotted lines indicate chance-level classification accuracy, and dashed lines indicate chance-level attention accuracy.

Across both datasets, the network is able to learn each task in isolation. However, for MNIST tasks involving partial supervision, indicated by dashed outlines in the figure, multitask learning provides a clear performance advantage. For example, in the symbolic-orienting task (trained only with cross-entropy loss, without supervision for attention maps) and the top-down search task (trained only with MSE loss, without classification labels), multitask learning improves both attention accuracy and classification performance. The results for COCO tasks are particularly interesting. In the recognition task, the network successfully learns where to attend despite receiving no explicit supervision for attention maps. In contrast, the top-down

search task fails to recover correct labels when trained in isolation, likely due to the task's inherently ambiguous label-to-object mapping. These results suggest that multitask training can provide helpful inductive structure, especially when supervision is partial or the task is under-constrained.

Here we would like to note that the attention maps for the single-tasks that were trained only through classification look reasonable, despite the low attention accuracy for the given ground truth (Fig. 2).

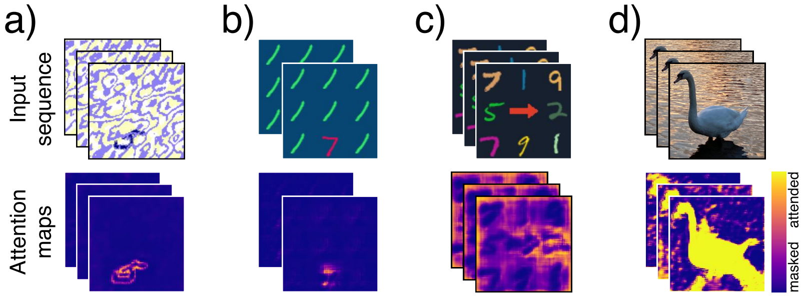

Supplementary Figure. 2. Attention maps for single-tasks trained only on classification. a) Object recognition (MNIST digit), **b)** Pop-out saliency (MNIST digit), **c)** Symbolic orienting (MNIST digit), **d)** Object recognition trained on MS-COCO animals. Digit images are adapted and modified from MNIST dataset ©LeCun, Cortes, and Burges [LeCun et al., 2010], available under a CC BY-SA 3.0 license.

Single-task pretraining, multitask transfer learning

To better understand the interplay between tasks in our multitask learning framework, we conducted a set of transfer learning experiments with two key questions in mind: (1) Does pretraining on single tasks facilitate future multitask training? (2) To what extent does this facilitation depend on the pretraining task or the downstream tasks?

Answering these questions provides insight into factors such as computational efficiency gains or costs, task interference or facilitation, the emergence of shared representations, and how performance scales with increasing task complexity. To explore this, we first trained our model (including the task-embedding layer) on a single task until learning plateaus. We then continued training the same model on all tasks simultaneously, including the pre-trained task. This procedure is repeated independently for the three COCO tasks (Fig. 3) and the seven MNIST tasks (Fig. 4).

The COCO results in Fig. 3 suggest several interesting and encouraging patterns. Models pre-trained on recognition or perceptual grouping tasks readily adapt to additional tasks with little to no degradation in performance. In contrast, a model pre-trained on the top-down search task does not show the same facilitation for recognition. Another notable observation is the speed at which pre-trained models learn new tasks. Finally, in all cases, the network retains performance on the original pretraining task, indicating no catastrophic forgetting under this regime.

The results for MNIST paint a different picture (Fig. 4). We selected three representative cases to illustrate that not all tasks are equally facilitative. Specifically, models pre-trained on IOR or tracking either lose performance on the original task or impede learning of new tasks. Additionally, both of these pretraining tasks interfere with the performance of visual search, even in a model that was originally pre-trained on visual search itself (Fig. 4, right column). These findings highlight that certain tasks may introduce interference dynamics in the multitask setting, depending on how their representations interact with others.

Based on the results shown in Fig. 3 and Fig. 4, we draw the following conclusions: 1) *Aligned tasks*: Some tasks facilitate each other, meaning that learning one can enhance or accelerate learning of the other. We refer to these as aligned tasks. Our results suggest that recognition and perceptual grouping fall into this category. 2) *Orthogonal tasks*: Other tasks appear to interfere with one another, such that learning one can hinder learning of the other. We refer to these as orthogonal tasks. Inhibition of return and top-down search appear to behave this way, suggesting that additional sub-networks or task-specific dynamics may be required to support them effectively. 3) *Backbone potential*: These results also point to the possibility of constructing a "backbone model" pre-trained on core tasks such as recognition and perceptual grouping. Pretraining on such foundational tasks may help improve and accelerate the learning of new, aligned tasks added later in the training process.

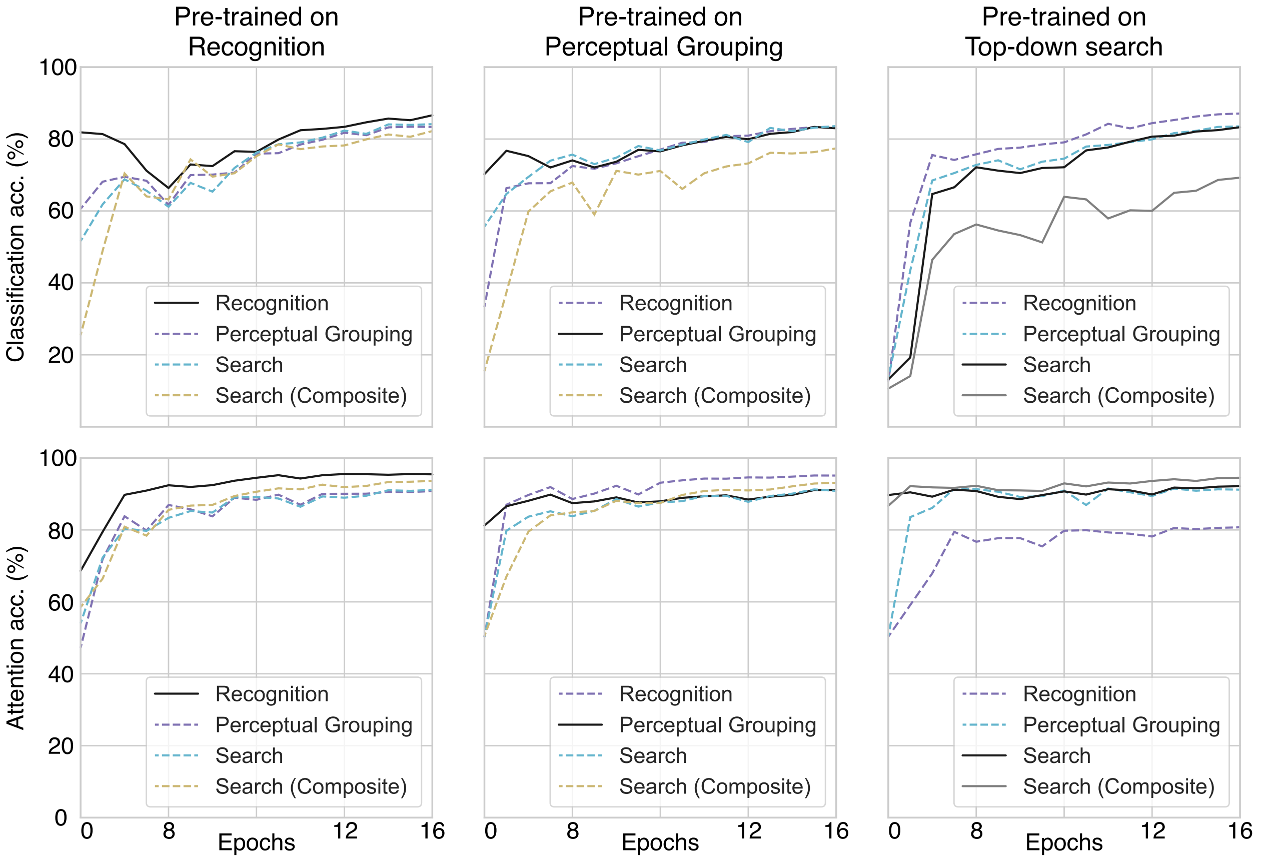

Supplementary Figure. 3. Multitask learning on a pre-trained COCO model. Attention and classification (validation) accuracy during multitask transfer learning for models pre-trained on single tasks. **left column)** Model pre-trained on the recognition task facilitates rapid learning of additional tasks, though classification accuracy initially drops. This dip may be due to the large learning rate at the peak of the warm-up schedule, as we use warm-up and cool-down learning rate scheduling. **middle column)** Model pre-trained on the perceptual grouping task enables faster and more effective multitask transfer learning compared to the other two tasks. This may be because the model received both classification and attention supervision during single-task pretraining. **right column)** Model pre-trained on top-down visual search does not yield effective transfer learning; classification accuracy for the composite search task and attention accuracy for the recognition task remain suboptimal.

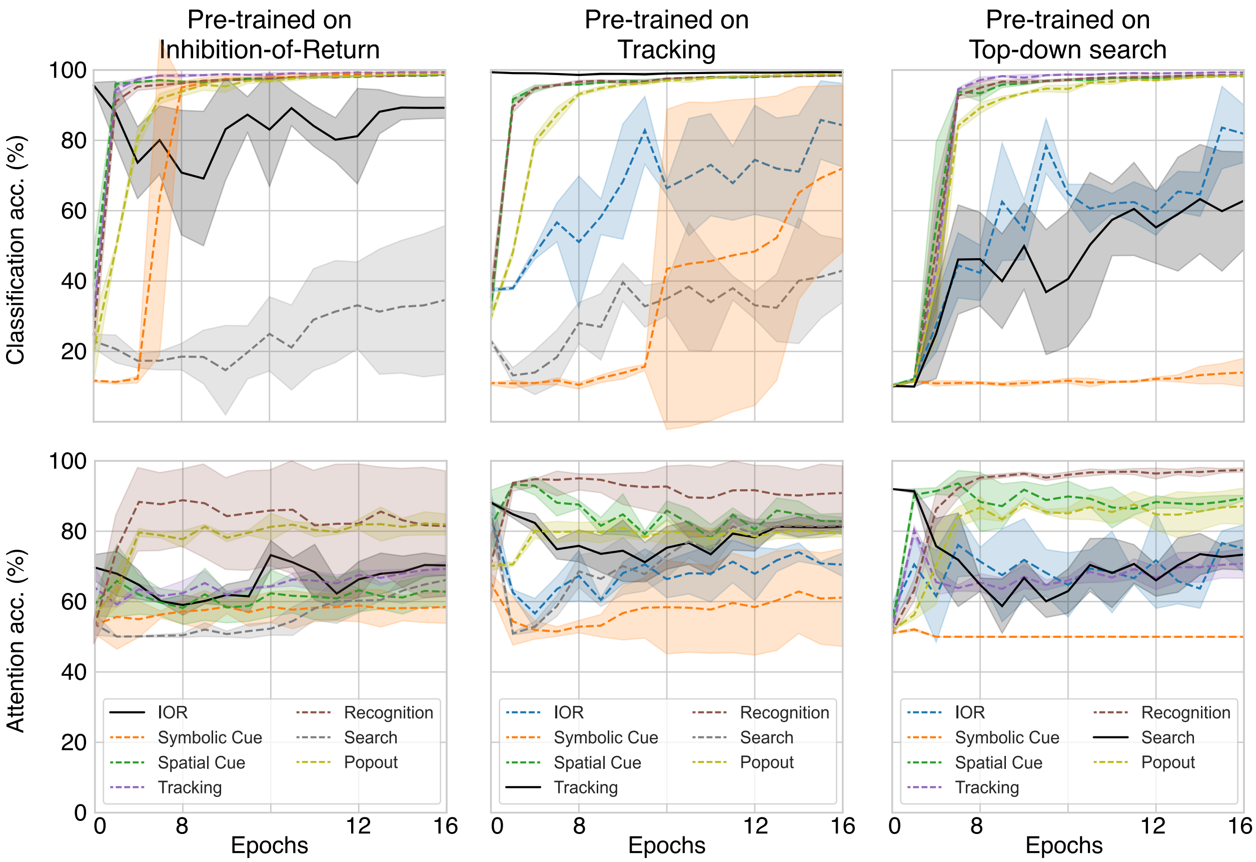

Supplementary Figure. 4. Multitask learning on a pre-trained MNIST model. Attention and classification (validation) accuracy during multitask transfer learning for models pre-trained on single tasks. **left column)** Model pre-trained on inhibition of return (IOR) task fails to accommodate multitask transfer learning, particularly for visual search task. **middle column)** Model pre-trained on object tracking does not enable strong transfer learning, although it retains performance on the original tracking task. **right column)** Model pre-trained on top-down visual search struggles to maintain its performance when new tasks are introduced.

Representational similarity analysis

Continuing our investigation of single-task versus multitask learning, a plausible concern is whether the network implicitly splits into separate, parallel single-task pathways during training. To evaluate this possibility and to assess whether shared representations emerge across tasks, we conducted a Representational Similarity Analysis (RSA; [Kriegeskorte et al., 2008]) using the Pearson correlation coefficient as the similarity metric.

For this analysis, we trained a model based on the bidirectional recurrent gating mechanism on three distinct tasks: (1) object recognition, (2) perceptual grouping via spatial cueing, and (3) top-down visual search. We used the STL-10 dataset [Coates et al., 2011], which comprises 10 image classes with 500 training samples per class, and a fixed subset of its validation set for evaluation. Since feature representations are spatially equivariant, we opted not to use the COCO dataset (we could not create image composites for the three tasks where the target object is always at the same location and same size). STL-10, with its smaller sample size, was chosen intentionally to increase the risk of overfitting, thereby providing a more sensitive test of whether truly shared representations emerge. It also provides us with control over location and size of target objects.

The trained model achieved classification accuracies of 70%, 74%, and 75% for object recognition, perceptual grouping, and top-down search, respectively. Attention accuracy reached 94% for perceptual grouping and 86% for top-down search. Notably, the high classification accuracy on the top-down search task, which was not explicitly trained using cross-entropy loss, strongly suggests that the network leveraged feature representations learned from the other two tasks, indicating the presence of shared internal representations.

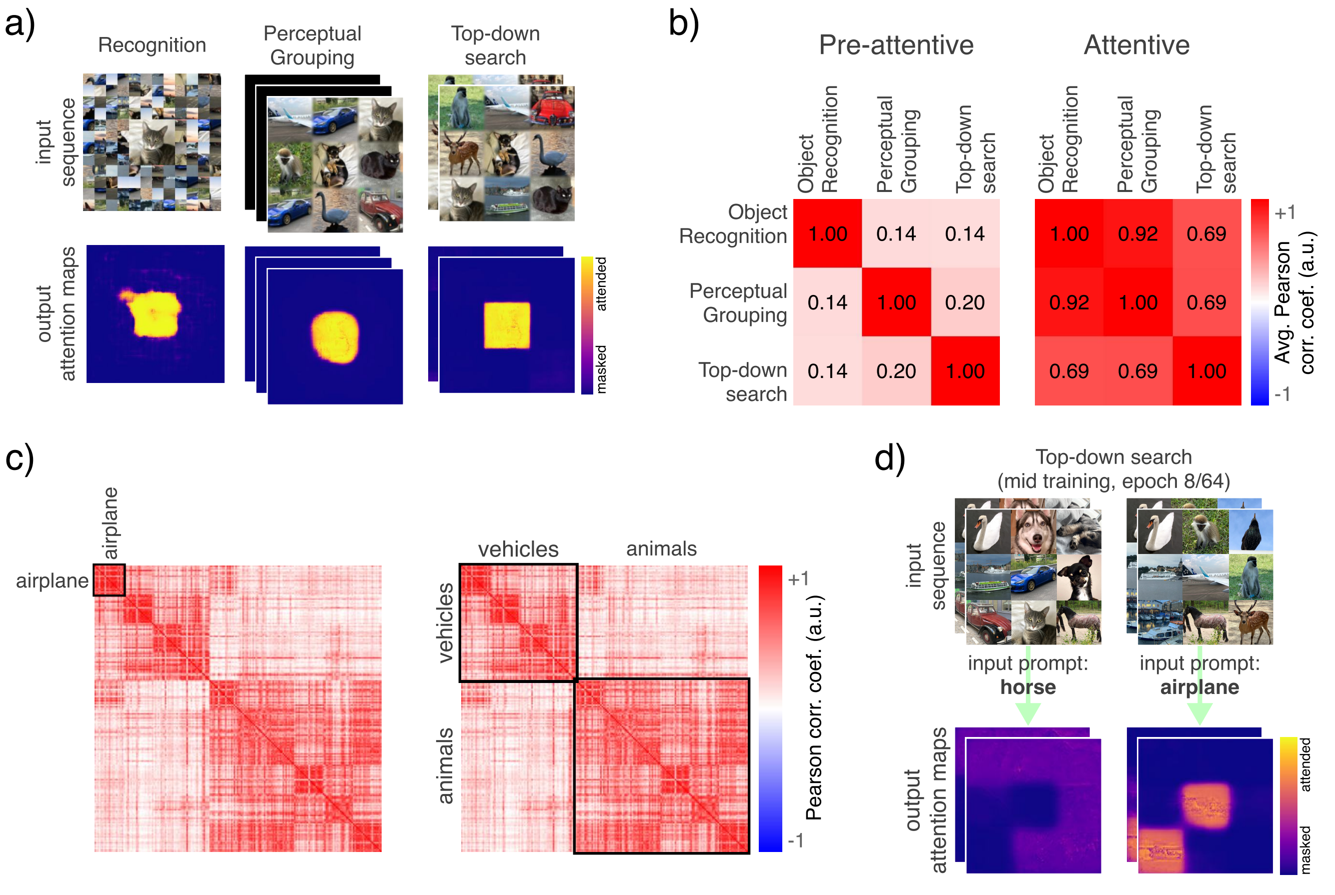

Supplementary Figure. 5. Representation similarity analysis. a) Example input samples used for RSA, constructed with target objects consistently positioned at the center of the image. **b)** Averaged cross-task similarity analysis of neural activity in the pre-attentive (left) and attentive (right) phases. The results are from the penultimate layer of the model trained on the STL-10 dataset composites. **c)** Attentive representation similarity matrix for 200 samples from the object recognition task, revealing strong neural correlation clusters at two levels: within the same class (left) and across the same superclass (right). **d)** Superclass correlations observed during training. Shown are output attention maps from the top-down search task for two validation samples at epoch 8, illustrating the model's early-stage ability to attend to relevant object categories despite not yet achieving high overall search accuracy (left: animals, right: vehicles).

The stimuli used for RSA were constructed such that the target object was always centered (Fig. 5a), although during training these objects were randomly positioned within composites. We first analyzed both pre-attentive and attentive neural activities (i.e., feature representations) for each input at the penultimate layer of the feature pathway. We then computed cross-task representational similarities for both attention phases across all three tasks and all input samples (Fig. 6). We expected lower cross-task similarities in pre-attentive representations (Fig. 6b, lower triangle) and higher similarities in attentive representations (Fig. 6b, upper triangle). Each entry in the similarity matrices represents the Pearson correlation coefficient between the feature representations of two samples from two different tasks in either the pre-attentive or attentive phase. To better quantify these trends and highlight the difference between attention phases, we averaged the diagonal values across all task combinations and samples (Fig. 5b). The results confirm our hypothesis: pre-attentive representations are largely dissimilar across tasks (Fig. 5b, left), while attentive representations show significantly greater similarity (Fig. 5b, right). This suggests that attention consistently suppresses irrelevant features and background activity across tasks, leading to shared task-invariant representations for the same target object. These findings further support the conclusion that the network leverages its attention pathway to solve a variety of tasks by dynamically aligning its internal representations toward the relevant stimulus features.

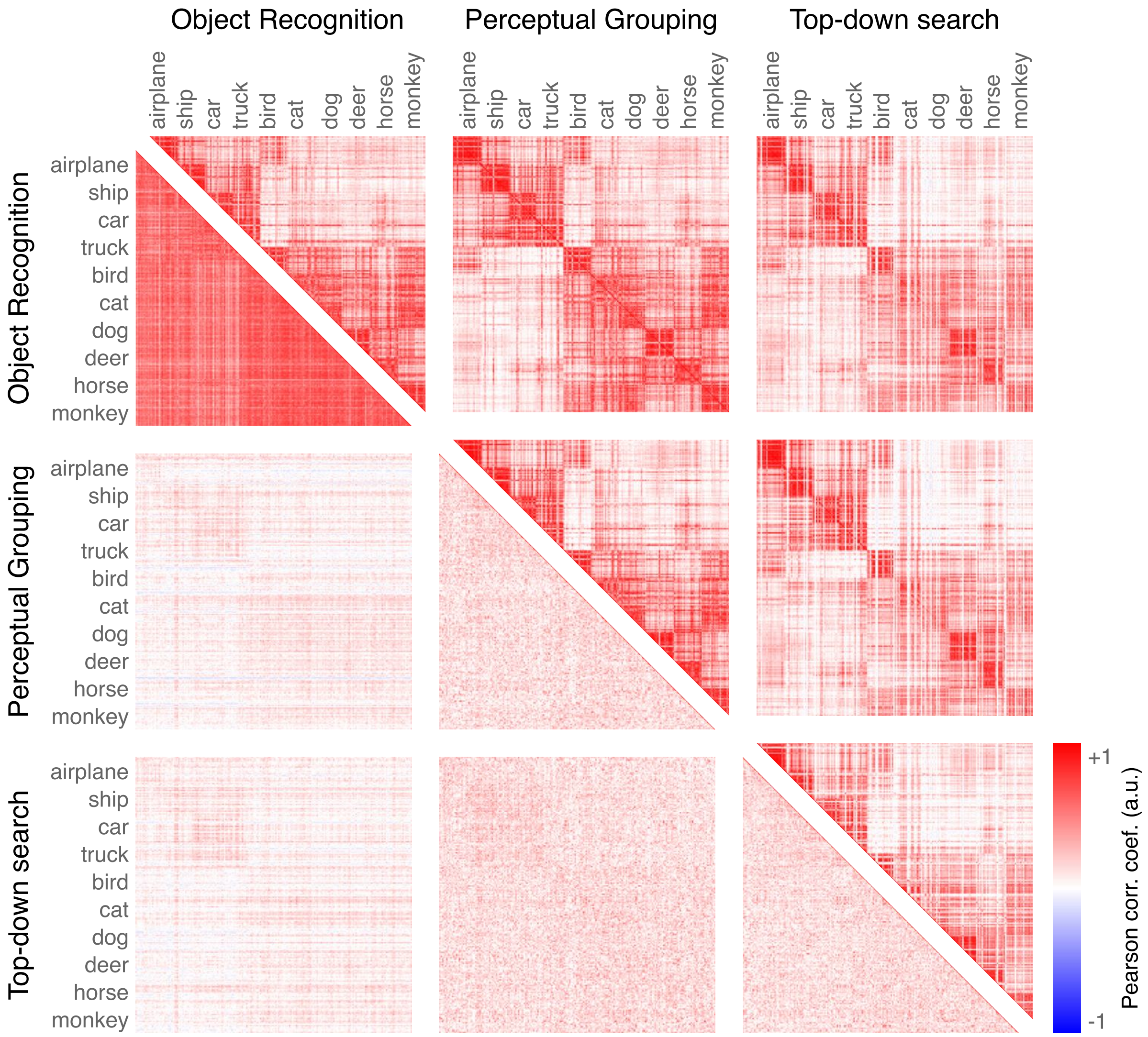

Supplementary Figure. 6. Representation similarity matrix. Results are from the penultimate layer of the model trained on the STL-10 dataset composites. Lower triangle: Cross-task similarity matrices of pre-attentive neural representations. Upper triangle: Cross-task similarity matrices of attentive neural representations. The observed increase in both auto- and cross-task correlations during the attentive phase strongly supports our hypothesis that the attention pathway is the primary mechanism for top-down neural modulation in our multitask paradigm.

Computational scaling with task count and iterations

To empirically evaluate how computational cost scales with task count and complexity, we conducted a controlled grid of training scenarios, systematically varying: (1) the number of tasks (from 1 to 5), and (2) the number of recurrent iterations per task (from 1 to 5). For this analysis, we used our COCO model (2 million learnable parameters). All experiments were run on a single NVIDIA A100 80GB GPU. Compute times were averaged over 10 epochs, each consisting of 32 mini-batches with 128 samples per mini-batch and trained on both CE and MSE losses. To ensure consistency, all reported times were normalized relative to the training time of the feature-path through a single feedforward pass (i.e., no recurrency and no attention) of 3.97 seconds. Our key findings are as follows: (1) Adding a second task increases compute time, but additional tasks beyond two have minimal impact (Fig. 7). This jump from one to two task is primarily due to the embedding layers introduced for multitask conditioning; and (2) Increasing the number of recurrent iterations per task (reflecting task complexity or temporal depth) results in sublinear growth in compute time. This suggests that our recurrent mechanism is computationally more efficient than a naive unrolling strategy.

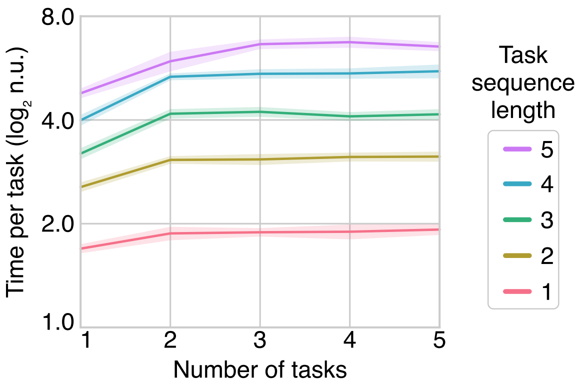

Supplementary Figure. 7. Computational scaling with number of task and iterations Normalized average training time per epoch per task in $\log_2$ scale as a function of number of tasks and number of recurrent iterations per task. While compute time for each task increases with the addition of a second task due to fixed overhead from task-embedding layers, it plateaus for three or more tasks. In contrast, increasing the number of recurrent iterations leads to a sublinear growth in compute time, indicating the efficiency of the recurrent gating mechanism over naive unrolling.

On the other hand, both CPU and GPU memory usage increase with longer sequence lengths due to the need to store intermediate recurrent states and gradients. It is also important to note that, in our current setup, input samples are dynamically generated during training (particularly for multi-object COCO scenes). This introduces additional CPU overhead that is not inherent to the model architecture and could be optimized separately.

In summary, while sequence length contributes more significantly to compute cost than the number of tasks, the system demonstrates reasonable scalability even as task count and temporal complexity increase.

Further experiments and analysis

Figure-ground separation

We trained a network based on bidirectional gating mechanism on a multi-modal classification task involving shape, color, and texture, without any attention supervision. Each input image contained a single object defined by three independent features: shape (triangle, square, circle, cross, hexagon, pentagram, heart, hexagram, and crescent), color (red, green, blue, yellow, cyan, and magenta), and texture (salt-and-pepper noise, soft-splotchy patterns, structured patterns, and solid fill). The object was randomly positioned on a background with color and texture drawn from the same feature distribution as the object (Fig. 8a). To ensure that figure-ground separation was always possible, we designed the dataset such that the object and background differed in at least one feature (i.e., color, texture, or both).

This setup allowed us to examine whether bottom-up processing alone (i.e., purely feedforward) is sufficient for this task, and whether top-down processing provides a functional advantage. We trained three versions of the network independently: (1) a feedforward model using only the feature pathway (i.e., a single forward pass) trained on samples from set (i); (2) an attention-based model incorporating both feature and attention pathways, also trained on set (i) and (3) a control condition in which a feedforward model was trained on set (iii), images without backgrounds (i.e., no figure-ground separation required). All models shared the same feature pathway architecture to ensure a fair comparison. Additionally, three specific feature combinations: triangle + red + salt & pepper, square + green + structured, and circle + blue + splotchy, were excluded from the training set to evaluate generalization.

Our results show that the attention-based model not only learns faster and achieves higher classification accuracy (Fig. 8b), but also generalizes better to unseen feature combinations (Fig. 8c). Moreover, despite being trained solely for classification, the attention-based network produces plausible and interpretable attention maps, even for novel, randomly generated shapes (Fig. 8a). Finally, the control case confirms that the inferior performance of the feedforward model is not due to hyperparameter choice, but rather the presence of distracting background features.

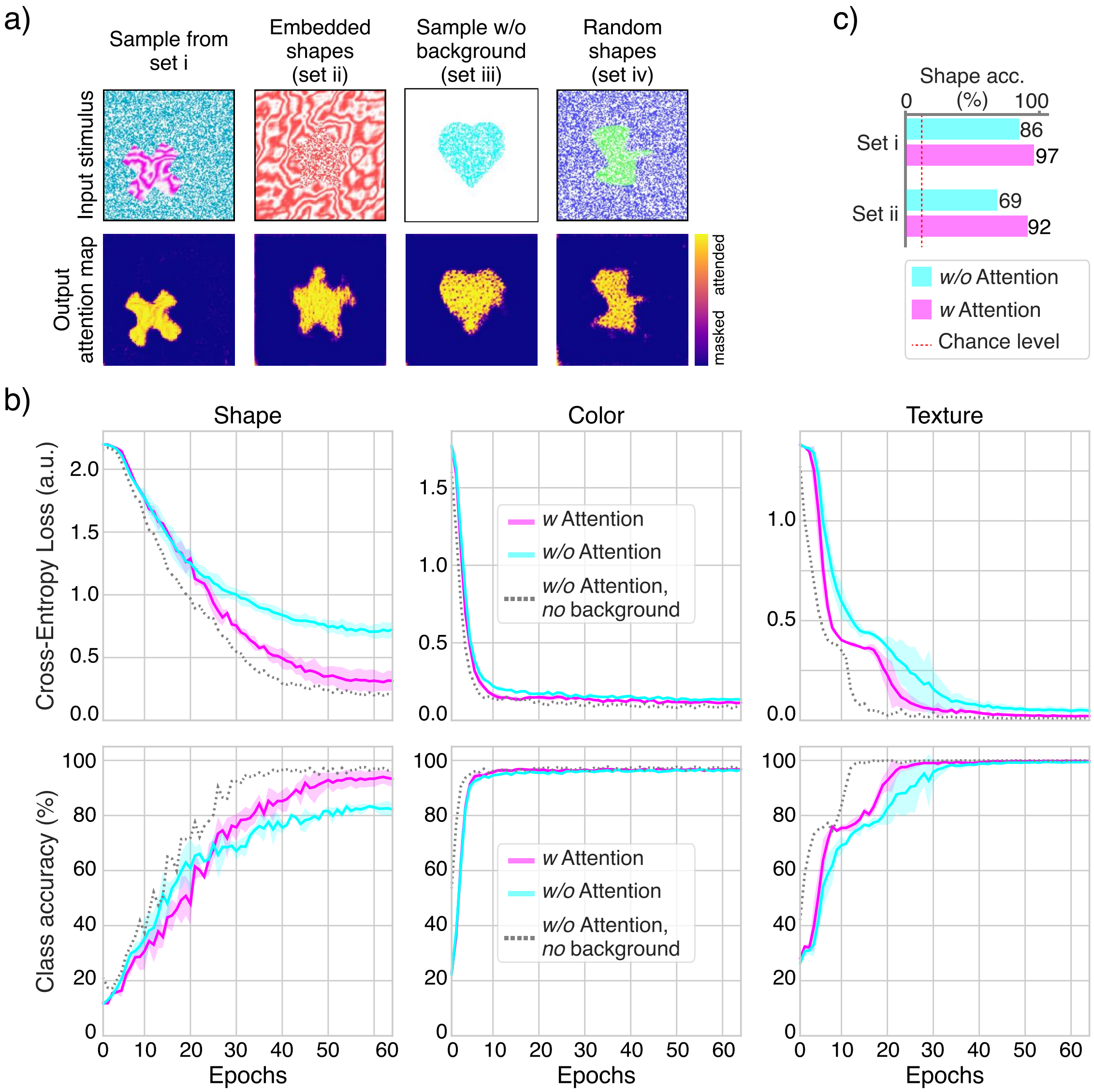

Supplementary Figure. 8. Multi-modal classification task. a) Sample composites (top row) and corresponding attention maps (bottom row) generated by the attention-based model. (Note: input image colors are inverted for better visualization.) The model is trained on samples from set (i). Set (ii) contains samples where the object and background share at least one feature (i.e., same color or texture). Set (iii) includes images without backgrounds. Set (iv) includes randomly generated novel shapes. **b)** Validation cross-entropy loss and accuracy during training. The attention-based model converges faster and achieves higher validation accuracy than the feedforward model trained on the same dataset. Dotted curves indicate performance of the feedforward model trained on the objects on blank background. **c)** Shape recognition accuracy across different test sets for models with and without attention. The attention-based network generalizes better to out-of-distribution samples and performs more robustly on ambiguous stimuli (set ii).

Classification tasks for CelebA attributes

A challenge in sex classification is the many correlated features that are present in the CelebA dataset. For example, wearing eyeglasses is strongly correlated with the male class, while having blonde hair is more common among the female celebrities in

the dataset. Here, we look at features that are localized and hence the learned attention maps are easier to interpret. We train separate models solely on binary classification of: *sex* (Fig. 9 a), whether the person is *smiling* (Fig. 9 b), whether the person has *blonde or black hair* (Fig. 9 c), and whether the person is *wearing eyeglasses* (Fig. 9 d). The results show that the network trained on smiling puts more attention to the mouth, lower cheeks, and chin (Fig. 9 b), while the network trained on eyeglasses seems to attend strongly to the mid-face and eyes (Fig. 9 d), Surprisingly, the model trained on hair color seems to attend not only to the hair but also the eyes (Fig. 9 c).

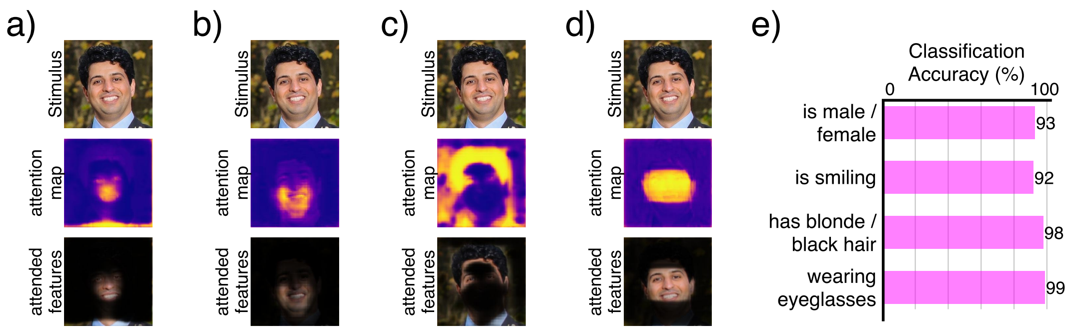

Supplementary Figure. 9. Binary classification for localized face attributes. Input image sequence, output attention map, and attended features for different models trained on binary classification of **a)** sex, **b)** smiling, **c)** hair color, **d)** and eyeglasses. **e)** Binary classification accuracy for different tasks.

Curve-tracing task

Training of the curve-tracing task is done entirely through the target attention maps (i.e., no classification error) (Fig. 10). We could successfully train the model to perform the task, with and without target curve supervision. The results shown in the main text are from the model that received target curve supervision (Fig. 10 a).

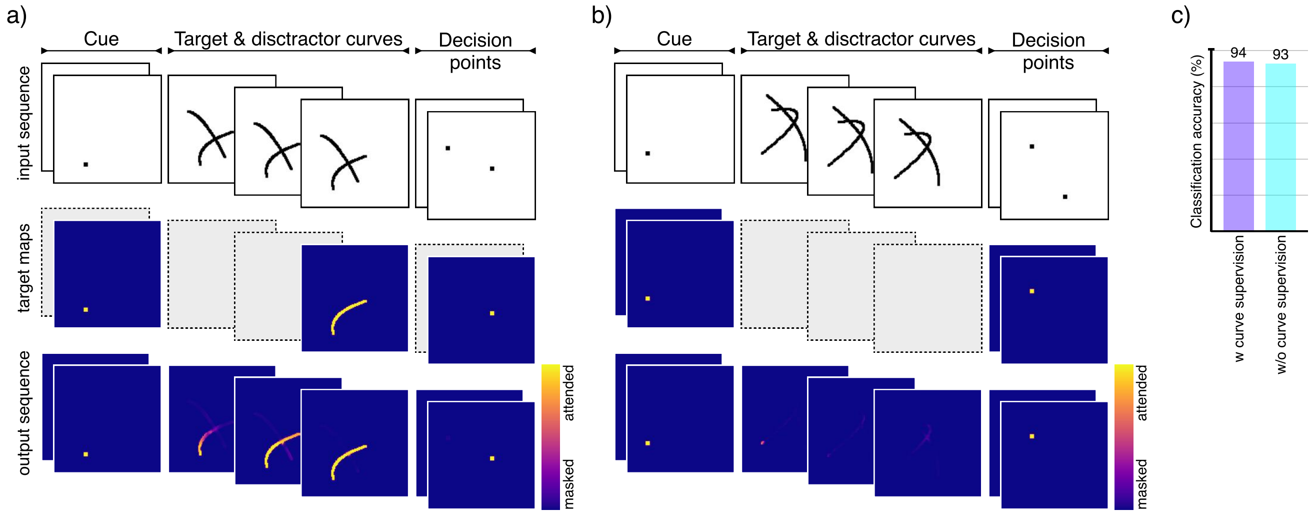

Supplementary Figure. 10. Training approach for the curve-tracing task. a) Full supervision: Target curve is provided while training on the task alongside the cue and target decision point. **b)** Partial supervision: Only the target attention maps for the cue and target decision are provided during the training, so the network has to learn about the curves and their objectness by itself. The gray dashed boxes imply what is NOT provided during training. **c)** Our model can learn to perform the task through either of the training regimes, although the partial supervision took longer to train.

There are some interesting observations to be made from this experiment. For example, the model seems to have an inherent attention (or grouping) propagation behavior (Fig. 11 a versus b) which could be further investigated to see if our model produces similar results to those from [Ekman et al., 2020] on top-down object-based attentional spread. Also, even though the model is really good in separating the two curves in most scenarios (e.g., Fig. 11 c), it fails to make a decision (i.e., choosing a single point) when the curves are ambiguously placed, and attends to both decision points (e.g., Fig. 11 d).

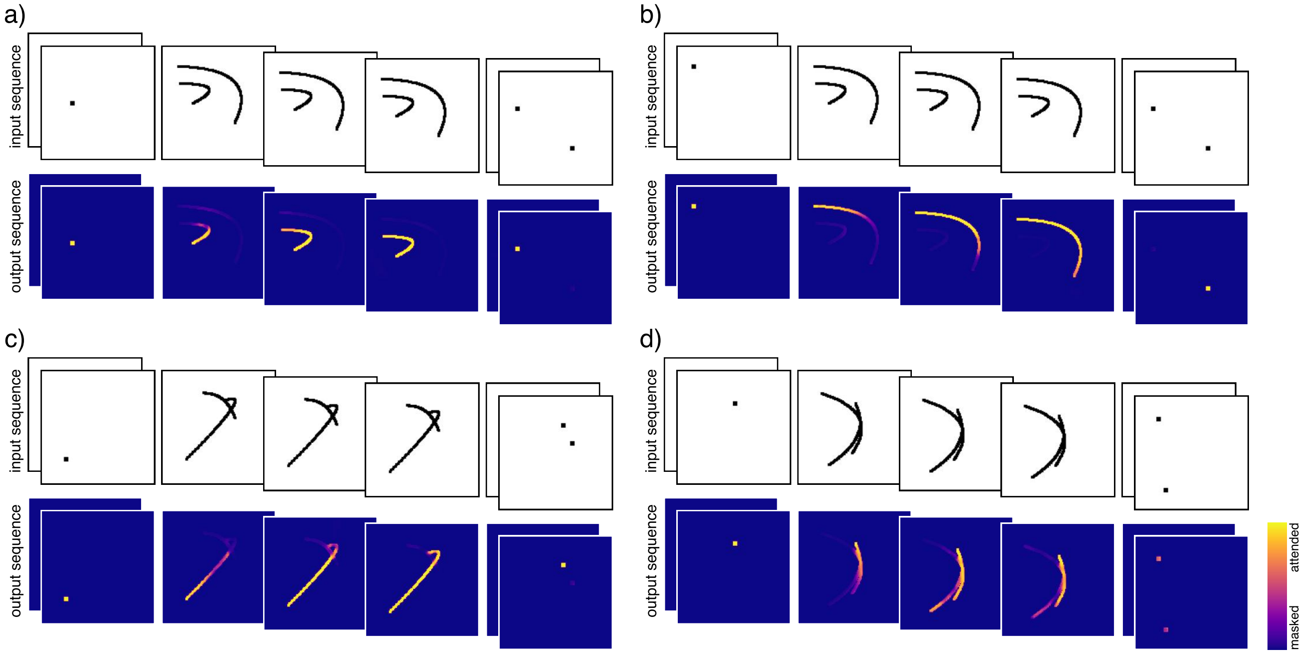

Supplementary Figure. 11. Multiple samples from the curve-tracing experiment. a-b) Stimuli and output attention maps for a short and for a long curve, showing an apparent presence of gradual attentional spread. **a)** Attention propagation on the short curve versus **b)** attention propagation on the long curve. **c)** Example of an ambiguous sample where the model makes the correct attention and decision. **d)** Example of an ambiguous sample where the model fails to trace the correct curve and make the final decision.

Attention improves learning

Our hypothesis is that feedforward neural networks (i.e., networks lacking an attention pathway) perform well when the foreground (target object) can be easily segregated from the background. In such cases, bottom-up feature integration is often sufficient to marginalize irrelevant background information. However, when the background shares similar features with the foreground, we expect feedforward networks to struggle with accurate classification.

To test this, we constructed several composite datasets derived from CIFAR-100 images [Krizhevsky, Hinton, et al., 2009], where the background for each target object is composed of patches from other images (Fig. 12a). The composite datasets are structured as follows: (Set i): The target image is randomly placed among 16×16 patches from randomly selected distractor images (Fig. 12i). (Set ii): Similar to Set i, but the target image itself is divided into four patches (without shuffling), and all patches (including the target) are visually separated by noise frames (Fig. 12ii). (Set iii): The background is composed of 1×1 pixel patches randomly sampled from eight distractor images, effectively creating a noisy background (Fig. 12iii). (Set iv): All background patches come from the same image, repeated eight times, resulting in a more coherent background (Fig. 12iv). (Set v): Spatial cues are presented before the main stimulus (for two iterations), while the composites could be taken from any of the previous sets (i–iv) (Fig. 12v). This set is only used with the attention-based model.

We trained three separate networks, each using the same backbone architecture and all trained solely for classification (i.e., no attention loss was applied, even for the model with attention pathway). The three training scenarios were as follows: (0) A control network without attention, trained on composites from Set iii (Fig. 12iii); (1) A second feedforward network, trained on composites from Set ii (Fig. 12ii); and (2) A network with attention pathway, trained on Set ii composites with a mix of samples both with and without spatial cues (Fig. 12ii, v). For validation and testing, we used composites from Set i to assess generalization under consistent conditions. Our results show that the network with attention not only converges faster during training (Fig. 13a), but also achieves higher classification accuracy and greater robustness to background noise (Fig. 13b). Moreover, the control condition confirms that the reduced performance in feedforward networks stems from the presence of ambiguous background features, rather than suboptimal hyperparameters (Fig. 13a).

Multi-modal top-down visual search

We trained a model based on the Bidirectional Recurrent Gating (BRG) mechanism to simultaneously perform recognition and top-down visual search across three modalities: shape, color, and texture. Each input prompt specifies one target class for each modality, resulting in a three-part search query for shape, color, and texture. Input images are composed of 16 objects

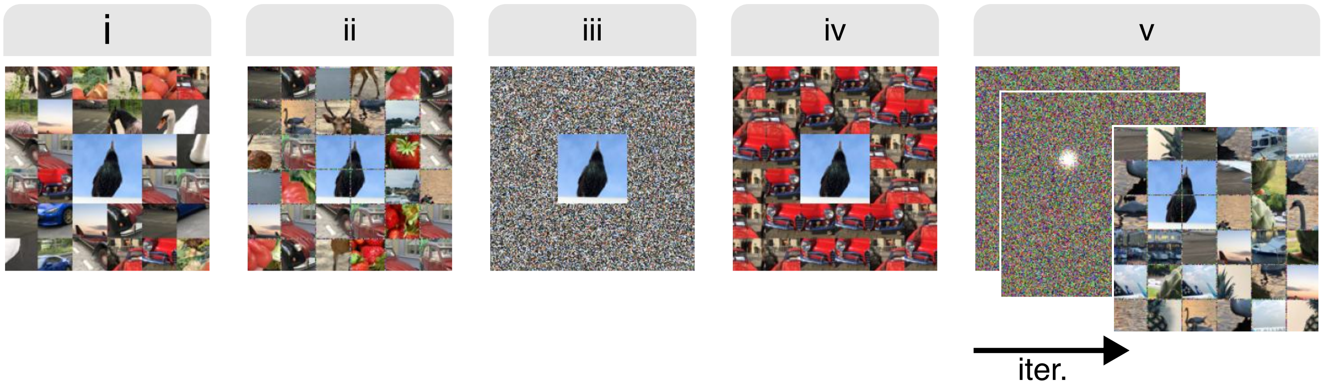

Supplementary Figure. 12. Image composites with varying background complexity. For better visualization, we used STL-10 images in the figure, as CIFAR-100 images are lower in resolution. Also, here the targets are centered in sample images, but during training and evaluation the targets were randomly placed. **i)** Target image is intermixed with large patches of random distractor images from the same dataset. **ii)** All the patches, including the target image, are visually separated with noise frames. **iii)** The patches of distractor images are of size 1×1, effectively creating a noisy background. **iv)** The patches of distractor images are from the same image, repeated 8 times. **v)** The image composite is preceded by two iterations of spatial cue.

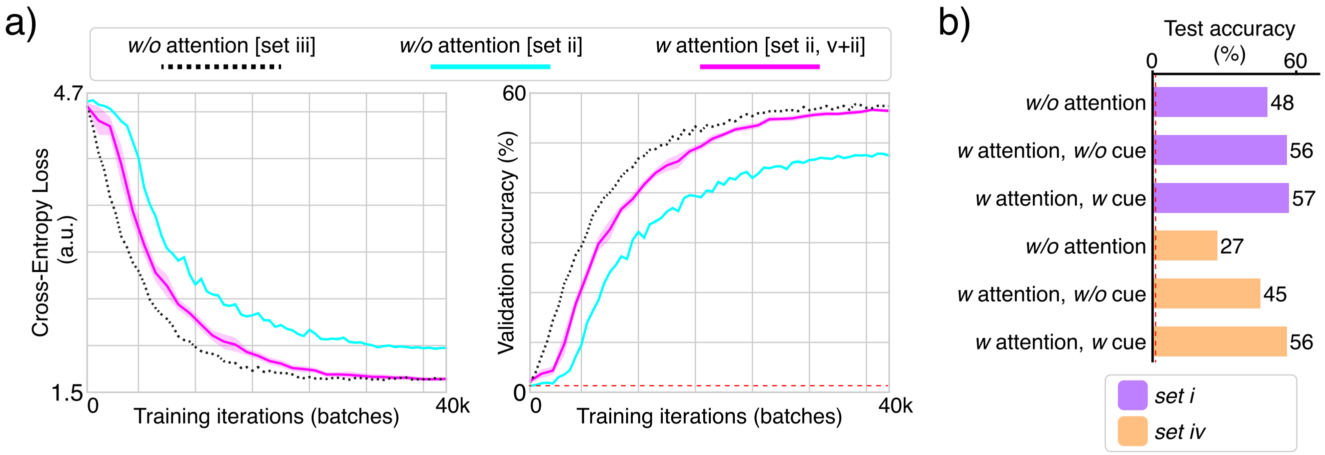

Supplementary Figure. 13. Attention improves learning. a) Validation accuracy and cross-entropy (CE) loss during training. The attention-based model trained on Set ii with a mix of samples both with and without spatial cues achieves higher accuracy and faster convergence compared to the model without attention. The black dotted line shows the performance of a control model (feedforward-only) trained on Set iii. Chance-level performance is indicated by the red dashed line. **b)** The attention-based model also generalizes better to out-of-distribution samples from Set iv.

arranged in a 4×4 grid (Fig. 14a). The shapes consist of nine distinct classes: triangle, square, circle, cross, hexagon, pentagram, heart, hexagram, and crescent. Colors are drawn from six categories: red, green, blue, yellow, cyan, and magenta. Textures are generated from three stochastic processes: Salt-and-pepper, sampled from a normal distribution, Soft-splotchy, generated via correlated noise, and Structured pattern, produced by repeating a randomly sampled kernel to form a pattern. Due to the randomness in texture generation, instances from the same texture class can vary significantly (Fig. 14b). While the full combinatorial space includes $9 \times 6 \times 3 = 162$ unique objects, our multi-modal approach reduces the effective search complexity. By concatenating the one-hot encodings of the three target categories into a single 18-dimensional prompt vector, the network performs targeted search across only $9+6+3 = 18$ individual feature classes. Each input image may contain zero, one, or multiple instances matching the specified combination of features. The model is trained to locate and attend only to objects that satisfy all three target properties (Fig. 14c–e).

For the top-down search task, the model is trained on three-iteration input sequences. The training objective is consistent with previous tasks: minimizing the cross-entropy loss for the recognition output and minimizing the mean squared error (MSE) between predicted and target attention maps, given both the input image and the associated prompt. The model achieves 99% accuracy in selecting the correct object during the top-down search task, with a precision of 94%. For the recognition task, the

network reaches 99% classification accuracy across all three modalities (shape, color, and texture) and 97% attention accuracy. To ensure generalization and examine against overfitting, three specific feature combinations "triangle + red + salt & pepper", "square + green + structured", and "circle + blue + splotchy" were explicitly excluded from the training dataset.

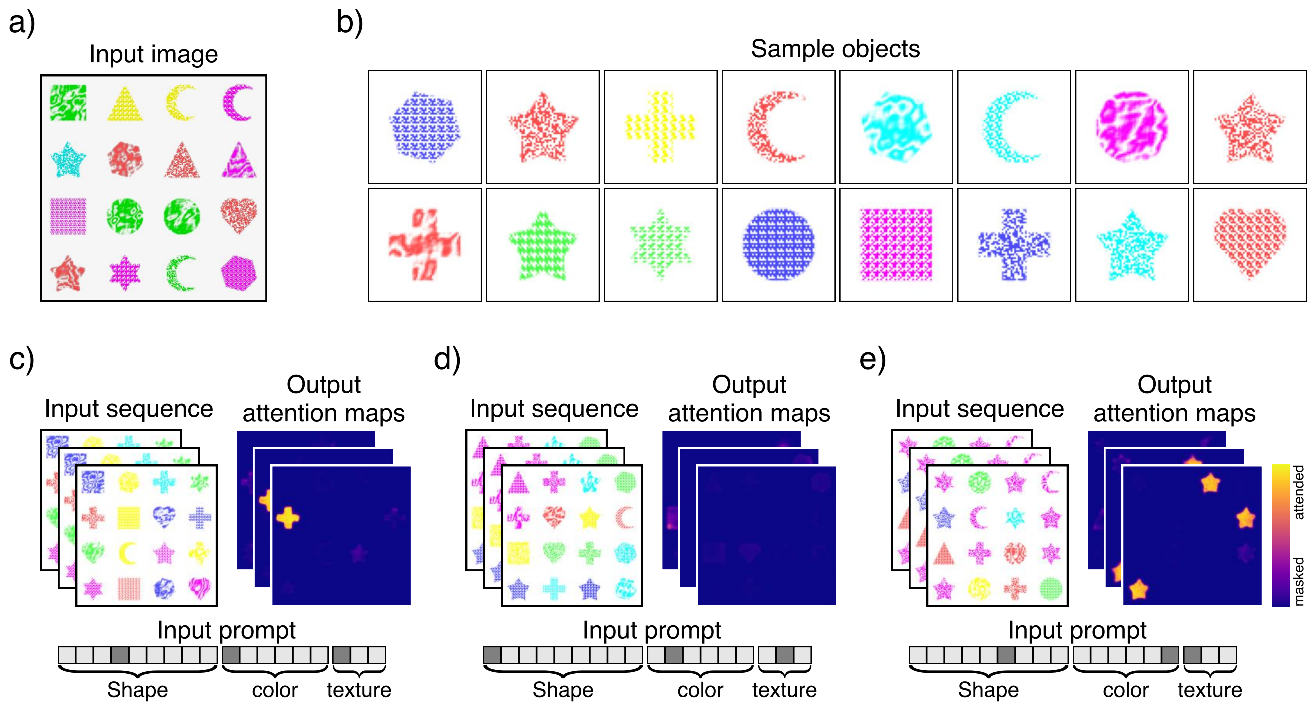

Supplementary Figure. 14. Top-down multi-modal visual search. a) Example input image for the multi-modal search task, composed of 16 objects arranged in a 4×4 grid. **b)** Each object is defined by a unique combination of shape, color, and texture. **c-e)** Three input-output examples. In each panel, input images are shown on the left, corresponding input prompts are shown below (schematic of a one-hot encoded input), and model's output attention maps are shown on the right. The input prompt is a concatenated one-hot vector encoding the target shape, color, and texture classes into a single vector. **c)** Illustrating a search example where only a single target object is present. **d)** Illustrating a search example where no target object is present. **e)** Illustrating a search example where multiple target objects are present. Note: Input image colors have been inverted for better visualization.

References

Coates, A., Ng, A., & Lee, H. (2011). An analysis of single-layer networks in unsupervised feature learning. *Proceedings of the fourteenth international conference on artificial intelligence and statistics*, 215–223.

Ekman, M., Roelfsema, P. R., & de Lange, F. P. (2020). Object selection by automatic spreading of top-down attentional signals in v1. *Journal of Neuroscience*, *40*(48), 9250–9259.

Kriegeskorte, N., Mur, M., & Bandettini, P. A. (2008). Representational similarity analysis-connecting the branches of systems neuroscience. *Frontiers in Systems Neuroscience*, *2*, 4.

Krizhevsky, A., Hinton, G., et al. (2009). Learning multiple layers of features from tiny images.

LeCun, Y., Cortes, C., & Burges, C. (2010). Mnist handwritten digit database. *ATT Labs [Online]. Available: http://yann.lecun.com/exdb/mnist*, *2*.
